# Regulation of Autophagy Via PERK-eIF2α Effectively Relieve the Radiation Myelitis Induced by Iodine-125

**DOI:** 10.1371/journal.pone.0076819

**Published:** 2013-11-05

**Authors:** Zuozhang Yang, Yongqing Xu, Lei Xu, Giulio Maccauro, Barbara Rossi, Yanjin Chen, Hongjun Li, Jing Zhang, Hongpu Sun, Yihao Yang, Da Xu, Xuefeng Liu

**Affiliations:** 1 Department of Orthopaedics, Kunming General Hospital of Chengdu Military Command, the Third Affiliated Hospital of Kunming Medical University, Kunming, Yunnan, P. R. China; 2 Department of Orthopaedic Oncology, Agostino Gemelli Hospital, Catholic University of Rome, Largo Francesco Vito 1, Rome, Italy; 3 Institute of Medical Biology, Peking Union Medical College, Chinese Academy of Medical Science, Kunming, Yunnan, P. R. China; Technische Universitaet Muenchen, Germany

## Abstract

Radiation myelitis is the most serious complication in clinical radiotherapy for spinal metastases. We previously showed that ^125^I brachytherapy induced apoptosis of spinal cord neurons accompanied by autophagy. In this study, we further investigated the mechanism by which ^125^I radiation triggered autophagy in neural cells. We found that autophagy induced by ^125^I radiation was involved in endoplasmic reticulum (ER) stress and mainly dependent on PERK-eIF2α pathway. The expressions of LC3II, ATG12 and PI3K were significantly suppressed in PERK knockout neural cells. Meanwhile, the expressions of phosphorylated-Akt s473 and caspase3/8 all significantly increased in neural cells transfected with a PERK siRNA and which enhanced apoptosis of neurons after ^125^I radiation. The results were consistent with that by MTT and Annexin-FITC/PT staining. In annimal model of banna pigs with radiation myelitis caused by ^125^I brachytherapy, we have successfully decreased PERK expression by intrathecal administration of the lentivirus vector. The apoptosis rate was significantly higher than that in control group and which deteriorated radiation myelitis of banna pigs. Thus, autophagy caused by ^125^I radiation was mainly as an attempt of cell survival at an early stage, but it would be a self-destructive process and promoted the process of apoptosis and necrosis radiated by ^125^I for more than 72 hours. The study would be useful and helpful to maximize efficiency of radiation therapy in clinical therapy.

## Introduction

Metastatic spinal tumors are the most frequent bone metastasis and they usually cause the destruction of vertebral bodies and accessories, even compression fractures, followed by spinal deformity and spinal instability [1], [2], [3], [4]. Spinal metastases seriously affect patient's life quality and psychological status by severe lesion pain and even neurological dysfunction. Radiation therapy is an important method for treatment of spinal metastases [5], [6]. It is a form of radiation therapy in which radiation is delivered directly inside or close to a tumor. The tumor tissue can accept highest dose of irradiation, while normal tissues and organs get very low exposure doses. So it is an effective way to kill tumor cells locally and protect healthy tissues. However, there still exist some side effects, such as radiation damage to the tissue around the seeds, which may cause complications, especially radiation myelitis [7], [8]. Radiation myelitis is the most serious complications in clinical radiotherapy, and the incidences occurred in 1.2% to 28.5% [9]. It may probably be related with the ray damage, vascular injury or autoimmunity, etc. The problems that how to reduce the side effects and what are the mechanisms remained unclear and need to be further explored.

In previous study, we had successfully established ^125^I brachytherapy induced radiation myelitis animal model [10], [11]. Shrinkage of cell membrane, increased mounts of lysosome, swelled mitochondria and autophagic vacuoles with double-membrane structure accumulated in spinal cord lesions were observed by electron microscopy. Thus, it was speculated that autophagy may play an important role in the process of ^125^I brachytherapy-induced neuronal cell death of the spinal cord.

Autophagy (or autophagocytosis) is the basic catabolic mechanism that involves in cell degradation of unnecessary or dysfunctional cellular components through the lysosomal machinery [12], [13], [14], [15]. Apoptosis-induced cell death accounted for 20% in programmed cell death. It is likely that mitochondrial related autophagy blocked mitochondria-dependent apoptotic pathway to delay apoptosis [16], [17]. In radiation therapy, radiation can induce autophagy in normal and cancer cells [18], [19], [20], [21], [22]. The molecular mechanisms responsible for the regulation of autophagy have not been completely elucidated, although biochemical analysis performed during the last few years has identified several proteins that participated in the regulation of this cellular process, such as target of rapamycin (TOR), the phosphorylated inositol triphosphate kinase (PI3K), GAI3 protein, amino acids and hormones etc [23], [24], [25], [26], [27]. It has been previously reported that endoplasmic reticulum stress (endoplasmic reticulum stress, ERS) PERK-eIF2 pathway may be important for radiotherapy-induced autophagy [24], [26].

In this study, we detected the variations of autophagy related genes and proteins and investigated mechanism of autophagy induced by ^125^I brachytherapy in spinal cord cells. Next, in the animal model, we further explored neural function and protein changes in Banna miniature pigs with radiation myelitis. The feasibility and efficacy of intrathecal injecting autophagy disturbance agent was evaluated in treating radiation myelitis. Therefore, it may be helpful to provide new ideas for clinical treatment of ^125^I seeds induced radioactivity myelitis.

## Materials and Methods

### Reagents

0.25% Trypsin in Hank's balanced salt solution, Neurobasal medium, B27 serum-free supplements, 10% FBS, and Trizol regents were obtained from Gibco. The monoclonal antibodis were as follows: PERK (Santa Cruz, sc-9477), eIF2a (Santa Cruz, sc-30882), anti-ATF4 antibody (Abcam, ab1371), anti-p-PERK (Thr980) (CST, 3179S), anti-p-eIF2a (CST, 9721s), anti-LC3II (CST, 2775S) ATG12 (C-18) (Santa Cruz, sc-70128), PI3K-p110γ (N-16) (Santa Cruz, sc-1404), goat anti-rabbit IgG-HRP (Santa Cruz, sc-2004) and goat anti-mouse IgG-HRP (Santa Cruz, sc-2005). Total RNA extraction kit (TaKaRa, MK700) and cDNA reverse transcription kit (TaKaRa, 6110) and Real-time PCR kit (TaKaRa, D323) were all obtained from TaKaRa.

### Radiation source and reagents

Brachytherapy seeds iodine-125 (BT-125-I) were purchased from Shanghai Xinke Medicine Ltd. (Shanghai Xinke Medicine; Sales No.: BTS10366A/BT10686A/BTS10993A). Apparent radioactivity was 1.00 mCi/seed and half time was 59.4 days. The validity period was 135 days. Before leaving the factory, ^125^I seeds were randomly picked up for activity testing to confirm the seed container integrity and apparent activity of seeds. In fact, one day before operation, we randomly selected 30 seeds to test the apparent activity, and found the actual dose per seed was between 0.64∼0.67 mci, and the associated uncertainty was 0.03 mci which met the requirements. X-ray computed tomography was from Siemens Company, Germany; DSA (Digital Subtraction Angiography) were from Philips Company, Netherlands; Treatment planning system (TPS) were from Hejie medical instrument company, China. CRC-15R calibrator was from CAPINTEC Inc, USA.

### Animals

12 healthy adult female Banna mini-pigs were selected for experiments. The animals were provided and raised by the Animal Center at Kunming Medical College. Their masses ranged from 20 to 25 kg (average 22.7 kg). The ethics committee of the Third Affiliated Hospital of Kunming Medical University approved this study. The mini-pigs adapted laboratory environment for one week before modeling. This housing facility is a barrier housing facility, and it has been in keeping with national standard “Laboratory Animal-Requirements of Environment and Housing Facilities”. The care of laboratory animal and the animal experimental operation have conforming to “Chinese Administration Rule of Laboratory Animal”, after careful CT scan and consideration, T13 level in spine of banna pig was selected as our ^125^I implanting target because it is easier to control and facilitate the whole operation. Two pigs were kept as age-matched normal control, which were selected to intrathecally inject with cell culture medium defined as group A. In group B and C, 5 pigs in each group were intrathecally injected with negative control siRNA inteferencing agent and PERK si-RNA interferencing agent, respectively. In both groups, eight brachytherapy seeds were implanted into the spinal dura mater at the T13 level. Radiation dosimetry was based on previous studies [10], [11]. The average radiation dose for each group was 16.76±0.32Gy and 17.02±0.58Gy. After operation, vertebrae, spinal cord and particle changes were monitored by MRI after 1 month, 3 and 6 months. The diet and mental state, weekly movement (especially the hind legs), tail wagging, and the stool urine were all observed, according to the modified Tarlov score of the American Spinal Injury Association (ASIA) classification method.

### Transfection

Three pairs of siRNA for PERK were designed by NM_031599.1 in NCBI database. The transfection efficiency was detected by qPCR, and the most effective siRNA was used. Neural cells (3×10^5^ cells per well) were seeded into 24-well plates. PERK-siRNA and NT-siRNA were transfected by means of lipofection using Lipofectamine 2000 (Invitrogen; cat. no. 11668–019). The stock concentration of siRNA was 20 μM, and the working concentration was 50 nM.

### Western Blot Analysis

Whole cells were collected and washed twice by cold PBS. Then, they were lysed with RIPA (Tris 50 mmol/L, NP-40 1%, NaCl 150 mmol/L, EDTA 1 mmol, SDS 0.1%). The protein concentrations were quantified using the Bradford assay. The samples were injected to SDS-PAGE and transferred electrophoretically onto a nitrocellulose membrane. The membrane was blocked with 5% nonfat milk in TBST (Tris-HCl 50 mmol/L, NaCl 150 mmol/L, 0.1% Tween), then incubated with rabbit monoclonal antibody for PI3K, ATG12, LC3II and *β*-actin. The goat anti-rabbit IgG antibody coupled with horseradish peroxidase was used as a secondary antibody. The resulting bands were detected using an enhanced chemiluminescence Western blotting detection system according to the manufacturer's protocol. *β*-actin was used as an internal reference and three independent experiments were performed.

### Reverse transcription and real-time PCR

Total RNA was extracted from cells using Trizol agent, and the process is carried out in strict accordance with protocols of the kit. The total RNA (1 mg) was used for a reverse transcriptional reaction, and cDNA was reverse transcribed by using Revert Aid First Strand cDNA Synthesis Kit (Fermentas, cat. No. K1621). The following primers were used:

1. GAPDH Primer F: 5′-GACAACTTTGGCATCGTGGA-3′
  R: 5′-ATGCAGGGATGATGTTCTGG-3′
2. PI3K Primer F: 5′-CAGTGGAGACAGTGCGAACGTA-3′
  R: 5′-TCGGCAGTCTTGCCGTAGAG-3′
3. ATG12 Primer F: 5′-CAGAAACAGCCATCCCAGAG-3′
R: 5′-GCCTTCAGCAGGATGTCAAT-3′
4. LC3 II Primer F: 5′-TGGGTACTGGGAATTGAACC-3′
  R: 5′-CAGGACCTCATTGCCCTTAG-3′.

The PCR conditions for each set of primers were 95°C for 1 min, followed by 40 cycles at 95°C for 15 s, 60°C for 45 s, and 72°C for 1 min. At the end of amplification, the results were analyzed by Data Assist ™ v3.0 Software (ABI) in relatively quantitative analysis. The mean values were used by the three independent experiments.

### Rat cord neural cells culture

The 14-day-pregnant rats were selected and primary and subcultured spinal cord neural cells were cultured as described by Virginia S [28]. The cells were centrifuged and collected at 1500 rpm, 15 min, and the supernatant was discarded. Neural cells were re suspended and the concentration was adjusted to 1×10^6^/mL. The cells (100 μL) were grown into polylysine coated 24-well plates, and cultured in 37°C, 30% CO_2_ incubator. The neural cells were adherent in each well after 4 hours. The neurons were cultured in petri dishes for 24 hours before they were treated with autophagy inducers, which were divided into three groups according to the particle irradiation dose (12 mCi and 24 mCi, 48 mCi). As described [29], 15 ^125^I seeds were distributed evenly in a 3 cm diameter of circumference, and the other seed was located at the center of the circle. The dishes were placed in the incubator 5 mm above the ^125^I seeds, and cultured for 5 days. The initial irradiation dose was 8.11cGy/h, 16.22cGy/h and 24.33cGy/h, and total absorption dose was 973.2cGy, 1946.4cGy and 2919.6cGy, respectively.

### Confocal Microscopy Analysis

The neural cells were counted and seeded on poly-L-lysine coated cell plate at the concentrations of 2×10^6^/well, and the cells were treated by ^125^I. Cells were washed with pre-warmed 1× PBS and fixed with 4% paraformaldehyde for 15 min at room temperature. Cells were then permeabilized in 1× PBS containing 0.1% Triton-X-100 at RT for 12 min. After 3 washes, cells were blocked in PBS containing 0.1% Tween 20 with 5% BSA for 1 h at RT, followed by incubation with primary antibodies of LC3-II in blocking solution overnight at 4°C (1∶200). The next day, after three washes, cells were incubated with appropriate Alexa Fluor 488/555/647-conjugated secondary antibodies in blocking solution for 1 h at RT. Cells were washed with 1× PBS 3 times and mounted using Prolong mounting reagent (Invitrogen, cat. No. P7481). Images were digitally acquired using the LSM 510 Meta confocal microscope (Carl Zeiss) and processed using MetaMorph software (v7.7.2).

### Flow cytometric analysis

After ^125^I, RA and PA treatment, the neural cells transfected by NT-siRNA and PERK-siRNA were washed in cold PBS and resuspended at a concentration of 5×10^6^/mL for analysis. Cells were washed twice with cold 1× PBS and fixed with 4% paraformaldehyde (without methanol) for 15 min on ice. After 2 washes, the cells were fixed with 80% iced ethanol, −20°C overnight. Then, the neural cells were washed twice with PBS, and were blocked in PBS containing 0.25% Tween 20 with 1% BSA for 30 min on ice. 1200 rpm,5 min, then the cells were resuspended with 100 uL PBS containing 1% BSA, followed by incubation with rabbit anti-rat antibodies of LC3-II (1∶300) in blocking solution overnight at 4°C (1∶200). The next day, after three washes, cells were incubated with FITC-labelled goat anti-mouse IgG for 30 min. 10 μg/mL PI and 0.1% RNaseA(Sigma, cat. No. R6513)was added. 30 min later, the samples were analyzed on a FACSCalibur cytometer using Cell-Quest software (BD Biosciences).

### Cell proliferation assay

The cell proliferation was performed by MTT assay as described earlier [30], [31]. Neurons were counted by hematocytometry, and a total of 2×10^3^ cells per well in 200 μL complete medium were seeded in a 96-well plate.18 hours later, the fresh medium was changed and the neurons were transfected by PERK-siRNA and NT-siRNA. After12 h, 24 h, 48 h and 72 h, cell proliferation experiments were performed with a minimum of 3 biological replicates, and cell growth curve was draw by the data.

### Annexin V-FITC/PI staining

The annexin V-FITC/PI staining kit (Roche Applied Biosciences, Indianapolis, IN, Cat. No. 11 858 777 001) was used for the detection of apoptotic cells. Assay was performed as the protocols.

### Statistical analysis

Experiments were performed in triplicate. Basic statistical analyses were performed using the statistical software SPSS 16.0 (SPSS Inc., Illinois, USA). The statistical significance of any difference between group means was determined by applying a two-tailed *t*-test. The difference was considered statistically significant if *P*<0.05.

## Results

### The expression of autophage-related proteins were highly promoted after ^125^I radiation

**Figure 1:**
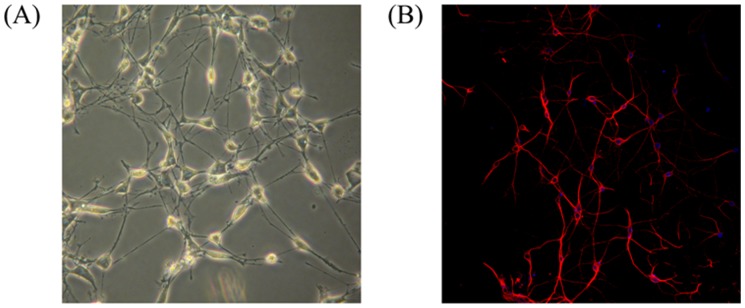
Neuronal cells culture and identification. The cells were cultured for 7(A) Cell morphology of cultured neuronal cells (the neurons photographed at 40× original magnification). (B) Immunocytochemical identification of neuronal cells marked by DAPI (blue) and TUJ1 (red) antibody (the picture photographed at 100× original magnification).

**Figure 2:**
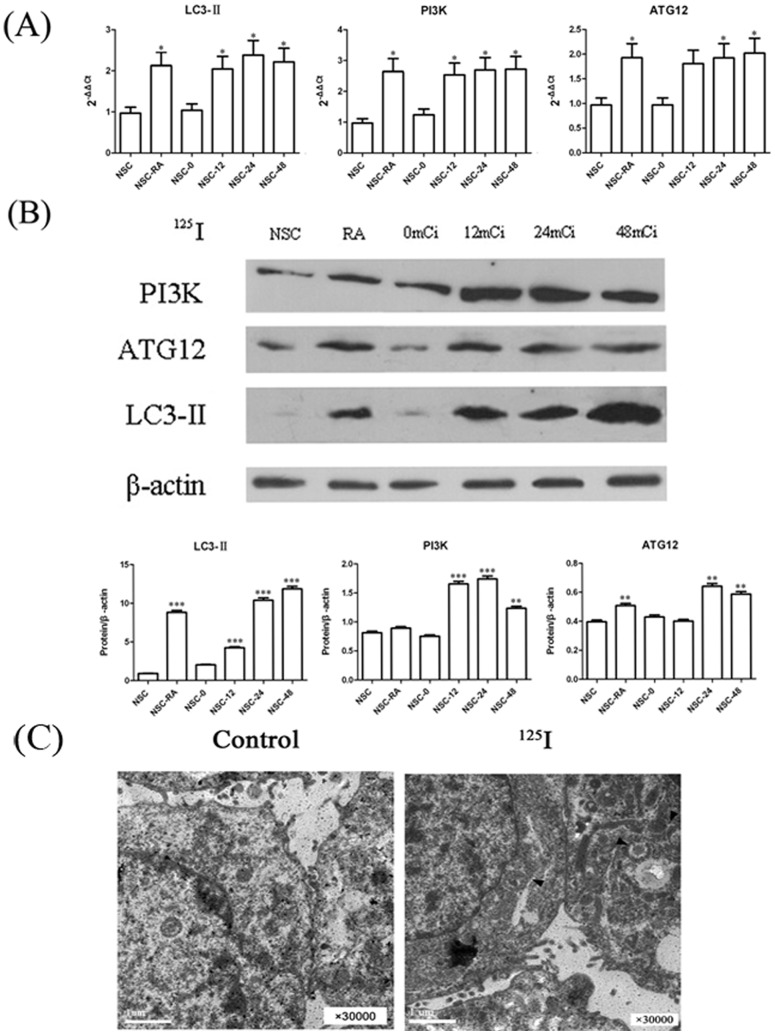
Induction of autophagy related proteins in neurons. (A) Dose response assay. Neural cells were treated with a range of ^125^I radiation (12mCi ∼48mCi) for 5 days. Relative mRNA expressions were measured by qPCR. The data were shown as mean value ± SEM., and they were representative of three independent experiments. (B) Neural cells were treated with a range of ^125^I radiation (12mCi ∼48mCi) for 5 days. Levels of proteins expression were measured by immunoblot ananlysis using antibodies against LC3II, Atg12, PI3K and beta-actin. Neuron cells treated with RAPA were as positive controls and untreated neurons were as negative controls. Results here were representative of three independent experiments. (C) Electron microscopic features of rat neural cells treated with ^125^I for 24 h. Arrows indicated autophagosomes.

Primary and subcultured neurons of rat spinal cord were generated as described in Material and Method. As shown in Figure 1, the data showed that >98% of the neurons were class III β-Tubulin (tuj1) positive cells. In order to detect the effects of ^125^I irradiation in neurons of rat spinal cord, total RNA of the neurons were extracted with different doses of ^125^I irradiation. As shown in Figure 2A, real-time RT-PCR results demonstrated that the expression levels of autophagy-related genes (LC3-II, PI3K and ATG12 etc.) were significantly higher than negative controls at 12mCi and the expression levels reached the peak when irradiation dose reached 24mCi. Consistent with the increased gene expression, western blot results showed that protein levels of LC3-II, PI3K and ATG12 increased in a dose-dependent manner (Figure 2B). Meanwhile, autophagosomes were clearly showed in electron microscopic photoes of rat neural cells treated with ^125^I for 24 h (Figure 2C).

### Autophagy induced by ^125^I radiation was mainly dependent on PERK-eIF2α pathway

**Figure 3:**
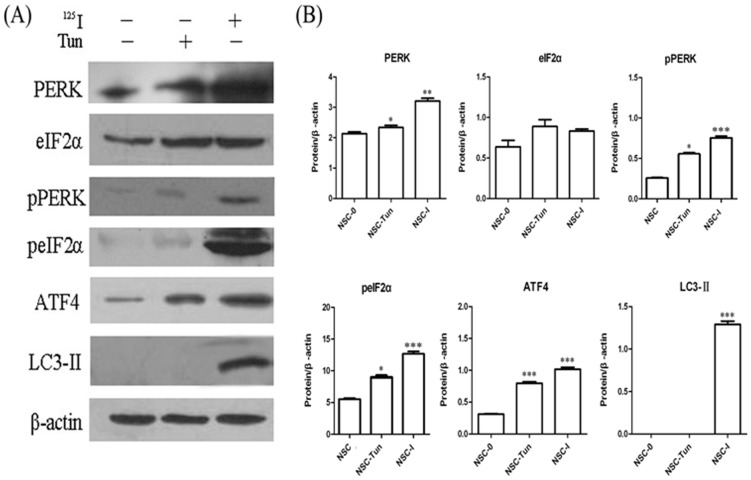
Induction of autophagy by ^125^I was dependent on PERK-eIF2α signal pathway. (A) Neural cells were treated with ^125^I for 5 days and tunicamycin (TUN) for 24 h. The levels of protein from PERK-eIf2α pathway were measured by immunoblot analyses. Results shown in each panel were representative of at least three independent experiments. (B) Quantification of the levels of protein from PERK-eIf2α pathway in neural cells following 24 h of treatment with tunicamycin (TUN) or ^125^I. Neuron cells treated with TUN were as positive controls and the untreated neurons were as negative controls (NSC-0). Data were representative of mean ± SEM. *, statistically significance in one-way anova test; *, *P*<0.05; **, *P*<0.005; ***, *P*<0.0005.

Based on previous *in vitro* results, the rat neurons were continuously exposed at 24mCi for 5 days. As demonstrated in Figure 3A, western blot analysis demonstrated that the expression levels of PERK, eIF2α, ATF4 and LC3-II highly increased. We also detected the phosphorylation levels of PERK and eIF2 after ^125^I irradiation. The results showed that phosphorylation levels of PERK and eIF2 were highly elevated at 24mCi for 5 days. Here, neural cells stimulated by tunicamycin were as positive controls and untreated neurons were used as negative controls. This was consistent with that by real time RT-PCR (Figure 3B).

### After^ 125^I irradiation, PERK-eIF2a-ATF4 signaling pathway was inhibited in PERK-siRNA group

**Figure 4:**
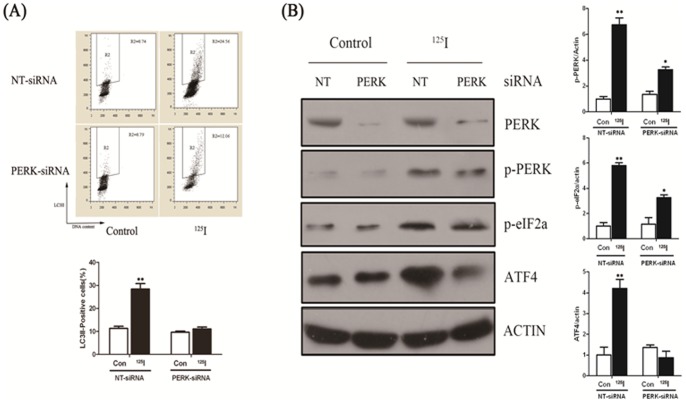
Neuron cells were transfected with RNAi against PERK and control (NT). (A) 16 h later, cells were allowed to recover for 3 days, and LC3II/DNA flowcytometry analyses after indicated period of time. The gate (R2) represented LC3II positive cells in each figure. * indicated statistically significant differences (*p*<0.05) between the levels of ^125^I treated and without treatment (NT) in cells transfected with PERK-siRNA or NT-siRNA. (B) Protein levels of PERK, p-PERK, p-eIF2α, ATF4 and *β*-actin were measured by immunoblot analysis in nueral cells transfected with RNAi specific to PERK and control cells (NT) which were radiated with ^125^I (24mCi) for 5 days. The expressions of p-PERK, p-eIF2α and ATF4 significantly decreased in response to ^125^I irradiation in neurons transfected with siRNA against PERK. Results were representative of three independent experiments. (B) Quantification of the mentioned protein levels in cells transfected with RNAi against PERK and control (NT) following ^125^I radiation. *, *p*<0.05, **, *p*<0.01, the error bar represented the SEM.

The data demonstrated that ^125^I induced autophagy was related to PERK-eIF2a signal pathway. Next, we designed three pairs of siRNA and the most effective siRNA was used to interfere with PERK expression with a final concentration of 50 nM in the experiment. We measured autophagy marker LC3II in PERK siRNA and negative control siRNA (NT-siRNA) transfected cells by flow cytometry analysis. As shown in Figure 4A, when irradiated by ^125^I, LC3II positive cells were significantly increased after 24 hours after transfected by NT-siRNA, which were much more than that transfected by PERK-siRNA. So, ^125^I radiation induced autophagy activity was suppressed in PERK knockout neural cells.

As shown in Figure 4B, the results by western blot analysis demonstrated that suppression of autophagy with a specific siRNA against PERK led to decreased PERK expression in the rat neural cells, compared with that transfected with NT-siRNA. However, PERK expression was not obviously decreased after irradiation by ^125^I. Interestingly, in the rat neural cells transfected with NT-siRNA, irradiation of these cells by ^125^I increased expression of p-PERK, p-eIF2a and ATF4. However, in PERK-siRNA transfected cells, the phosphorylation levels of PERK and eIF2a showed no significant differences between irradiated and non-irradiated cells and the expression of effector protein ATF4 enhanced slightly in the ^125^I irradiation group than that of non-irradiation. Similar results were seen following administration of tunicamycin (TM), a well known ER stressor.

### Autophagy related gene expression levels were not expressed in PERK-siRNA group

**Figure 5:**
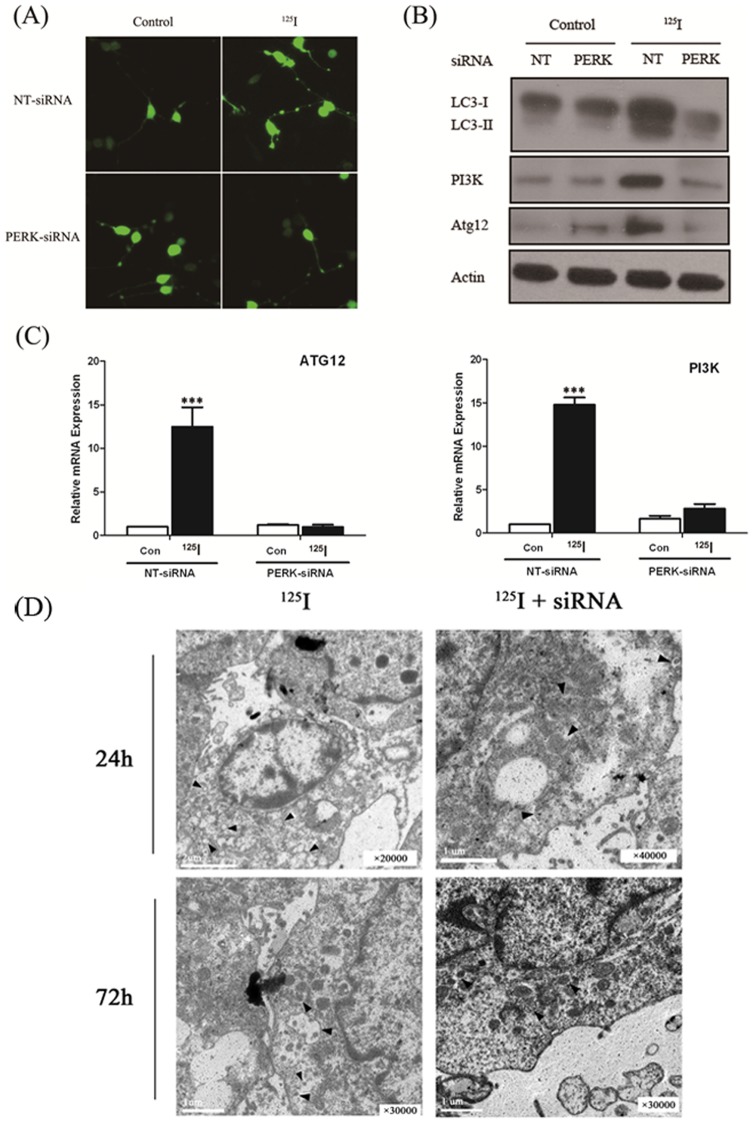
The proteins for the induction of autophagy in neuron cells under ^125^I radiation. (A) ^125^I-radiated neuron cells were transiently transfected with GFP-LC3 expressing plasmid. GFP-LC3 punctate formation was observed under confocal fluorescence microscopy in the absence or presence of PERK-siRNA. (B) Neuron cells transfected with RNAi for PERK and control (NT) were radiated with ^125^I for 3 days. Protein levels were measured by immunoblot analysis using antibodies against LC3II, PI3K, Atg12 and *β*-actin. (C) Neurons transfected with RNAi against PERK and control (NT) were radiated by 24mCi ^125^I for 3 days. Total RNA was isolated and levels of PI3K and Atg12 were analyzed by qPCR. n = 3; ***, *p*<0.0005. There was significantly different in two-tailed Student's *t* test; Column, mean; bars, SEM. (D) Transmission electron microscopic ultrastructures of the Rat neuron cells after ^125^I radiation for 24 h and 72 h. The ^125^I/siRNA combination significantly reduced autophagy, comparing with the ^125^I group alone. Arrows indicate autophagosomes.

In order to demonstrate that autophagy is induced during ^125^I irradiation, we next examined the autophagic bodies by confocal fluorescence microscopy. As shown in Figure 5A, punctate GFP-LC3 expression was significantly decreased in PERK knockout neural cells, compared to negative control cells after ^125^I irradiation. That means autophagic bodies were remarkably reduced in PERK-siRNA transfected neural cells compared with NT-siRNA transfected neural cells after ^125^I irradiation. We also found that in PERK-siRNA transfected neural cells, the number of autophagic bodies irradiated by^ 125^I did not obviously change compared with that without ^125^I irradiation (Figure 5A). That was consistent with the results of transmission electron microscopic ultrastructures in the rat neuron cells after ^125^I radiation for 24 h and 72 h, which obviously demonstrated that ^125^I/siRNA combination significantly reduced autophagy (Figure 5D). The expressions of ATG12 and PI3K were also detected by qPCR, amd the results revealed that there was no significant variation on the expressions of ATG12 and PI3K after ^125^I irradiation in PERK-siRNA transfected neural cells (Figure 5C). Here, the rat neurons transfected with NT-siRNA were used as negative controls and the expressions of LC3II, ATG12 or PI3K were at the background levels. However, in NT-siRNA transfected neurons, the expression of LC3II, ATG12 and PI3K were significantly elevated after ^125^I irradiation. We also examined protein levels of LC3II, ATG12 and PI3K by western blot (Figure 5B). As expected, the results of protein levels were consistent with that of real time PCR. Thus, we found that after ^125^I irradiation, autophagy could be activated through ER stress in rat neural cells.

### Autophagy inhibition enhanced apoptosis within 72 hours

**Figure 6:**
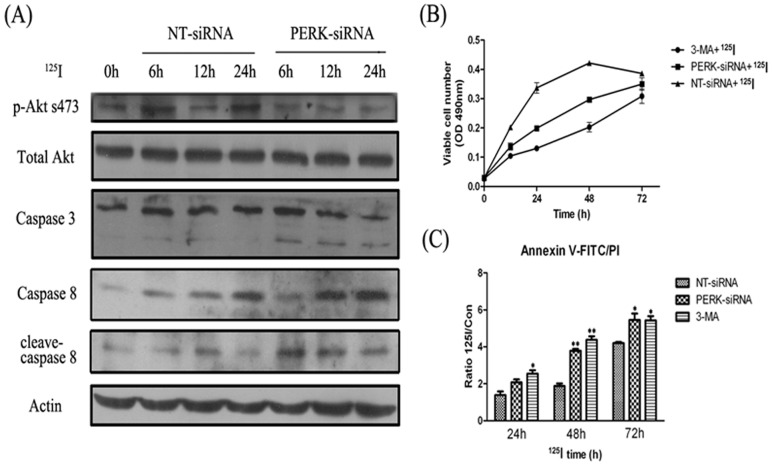
The relation of apoptosis and autophagy in survival of neuron cells radiated with ^125^I. (A) Neuron cells were transfected with RNAi against PERK and control (NT). After 16 hours, cells were cultured under ^125^I radiation for 6, 12, and 24 h, as indicated, or with no radiated with ^125^I (0 h). Equal amounts of the protein lysates prepared from the cultured cells were separated by SDS–PAGE, and the levels of AKT, p-AKT, PI3K, Caspase 3, Caspase 8 and actin were measured by immunoblot analysis. (B) Cells proliferation assay. Neuron cells were cultured and transfected with RNAi against PERK and control (NT). After 16 hours, cells were cultured and recover for 3 days, then treated with ^125^I and 3-MA (100 nmol/L). Proliferation was measured by MTT assay following instruction. Results here represent three independent experiments. Points, mean of three similar experiments (n = 3); bars, SE. (C) FACS analysis was performed. Neuron cells were stained with propidium iodide (PI) and Annexin V-FITC. *, *p*<0.05, **, *p*<0.01, the error bar represented the SEM.

^125^I irradiation related ER stress may induce apoptosis or autophagy through PERK signaling pathways. The results showed the variation of kinases in apoptosis related signal pathway by western blot analysis. As shown in Figure 6A, phosphorylation levels of Akt (s473) were decreased when PERK was interferenced for 12 to 24 hours, and increased the cleavages of pro-caspase 3 and pro-caspase 8, suggesting that apoptosis pathway was activated by inhibiting ^125^I radiation-induced autophagy. Next, we measured neuron cell viability by MTT (Figure 6 B). After ^125^I radiation, the neural cells transfected with NT-siRNA were more numerous than that transfected with PERK-siRNA and treated by3-MA 48 hours later (*p*<0.05). However, between 48 and 72 hours, neural cell proliferation was gradually slowing in NT-siRNA transfected group. There was no statistical difference with that in PERK-siRNA transfected group or 3-MA treated group after 72 hours by ^125^I radiation. We also detected the apoptosis of neural cells in different groups by annexin V-FITC/PI staining analysis, and the results were consistent with that detected by MTT (Figure 6 C).

### Evaluation of PERK-siRNA effects in radiation myelitis animal model caused by ^125^I brachytherapy in banna pigs

**Figure 7:**
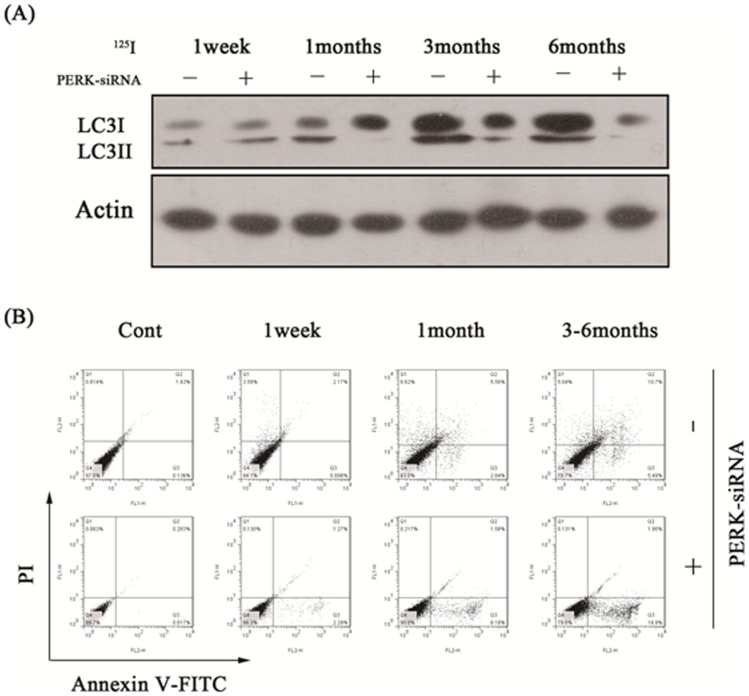
The motor fuction changes between control and PERK-siRNA groups in each time point under ^125^I radiation. (A) Porcines transfected with RNAi for PERK and controls were radiated with ^125^I for 6 months. Protein levels were measured by immunoblot analysis using antibodies against LC3II and β-actin at each time point of 1 week, 1 month, 3 months and 6 months. (B) Annexin V-FITC/PI double staining analysis at different times. The lower left quadrant (low-fluorescence PI and FITC signals) contains normal viable cells and the lower right quadrant (low-fluorescence PI and high-fluorescence FITC signals) defines cells in the early stages of apoptosis. The two upper quadrants (high-fluorescence PI signal) define dead cells. The numbers indicate the percentages of the different populations of cells.

In our previous studies, we have successfully established radiation myelitis animal model caused by ^125^I brachytherapy in banna pigs. One of the groups was successfully reduced PERK expression by intrathecal administration of the lentivirus vector (PERK-siRNA group), the other was control group with normal PERK expression that was by intrathecal administration of medium (Control group). We detected the expression of autophagy marker LC3II by western blot assay, and the results showed the PERK expression was significantly inhibited within 1 to 6 months after injection. Apoptosis and autophagy rates were also detected by FACS assay using Annexin V-FITC/PI double staining analysis. As demonstrated in Table 1 and Figure 7B, apoptosis rates were significantly higher than that in control group. Consistent with the results, we also observed more serious histological impairments and moving difficulty for banna mini-pigs in PERK-siRNA group.

**Table 1:** Apoptosis and autophagy rate between control and PERK-siRNA group both treated with ^125^I radiation in different times.

Time	N	Control		PERK-siRNA	
		Apoptosis rate Autophagy rate		Apoptosis rate Autophagy rate	
Control	10	0.139±0.232	0.615±0.102	0.053±0.020	0.094±0.011
1 week	10	0.127±0.018	3.376±0.360	2.676±0.217	0.141±0.024
1 month	10	2.424±0.222	8.984±0.390	7.942±0.533	0.237±0.026
3 months	10	6.200±0.444	5.384±0.396	17.614±0.756	0.179±0.021
6 months	10	6.694±0.248	4.842±0.248	18.230±1.715	0.258±0.046

## Discussion

The most common complication of radiotherapy in the treatment of metastatic spinal tumors is radiation myelopathy, which is caused by radiation damage and results in neuron apoptosis and necrosis. We previously found the autophagosomes in a banna mini-pig model of radiation myelitis. The purpose of this study was to explore the effects and mechanism of autophagy on radiation myelitis induced by ^125^I brachytherapy. Our findings revealed that the expression of autophagy related proteins significantly increased by PERK-eIF2α pathway of ER stress after ^125^I radiation, and sustained proliferation of the neurons of rat spinal cord within 48 hours. Autophagy serves a protective role in neural cells at an early time, however, it played an destructive role for neural cells after 72 hours after ^125^I radiation.

As we known, lipidated LC3-II, tightly associated with the autophagosomes and autophagic activity, which was used as a reliable marker for autophagy [32]. In our research, GFP-LC3 punctate formation was observed under confocal fluorescence microscopy after ^125^I irradiation, and the results demonstrated that ^125^I irradiation activated the autophagic activity in neurons of rat and this was consistent with that induced by 50 nmol/L of rapamycin [33]. The rapamycin, IFN-γ and starvation were all used as positive controls to induce autophagy. Meantime, the results also revealed that the autophagy-related proteins significantly increased when the irradiation dose was 24mci. So, 24 mci was the proper irradiation dose in our experiment.

We demonstrated that autophagy could promote the proliferation of neurons within 48 hours after ^125^I radiation, and was mainly as an attempt of cell survival. But after 72 hours of ^125^I radiation, autophagy played a destructive role in neural cells, for there were no significant differences among NT-siRNA treated group and 3-MA treated or PERK-siRNA treated groups on MTT assay and FACS assay. This was consistent with that when stress was excessive or unable to resolve, autophagy become a self-destructive process and promoted the process of apoptosis and necrosis [34].

Several studies have shown that autophagy was associated to ER stress which antagonized aggregation of misfolded proteins. Translated initiation factor eIF2α at serine 51 (S51) was phosphorylated by activated PERK to inhibit unfolded protein translation, but at the same time it induced translation of specific mRNAs encoding proteins that contributed to the adaptation process, such as the activating transcription factor 4 (ATF4) to alleviate ER stress. Also, PERK and eIF2α overexpression further increased radiation-induced autophagy and radiosensitivity. Interestingly, our study suggested that the levels of PERK and eIF2α were up-regulated and autophagy was activated after ^125^I radiation in neurons transfected by NTsiRNA (Figure 4B). Moreover, the autophagy related proteins PI3K and Atg12 were also upregulated in neurons after ^125^I irradiation. However, when autophagy was suppressed by 3-MA or PERK siRNA in neural cells, the expression of autophagy related proteins markedly was down-regulated, and apoptosis ratio was significantly enhanced compared to the negative control siRNA treatment group, suggesting autophagy could protect neural cells from death through antagonizing apoptosis induced by ^125^I irradiation. We also observed that transfected with effective PERK siRNA in neurons, PI3K activity and the expression of pAkt-S473 markedly decreased after ^125^I radiation suggesting that apoptosis of neurons was activated by blocking PI3K/Akt signaling pathway. Thus, when the neurons were irradiated by ^125^I for a long period of time (more than 72 hours), autophagy were unable to protect the cells to survive. Caspase-3, in addition to its role of apoptosis executioner, may prevent protection by autophagy and accelerate cell death by PI3K/Akt signaling pathway. Nevertheless, autophagy and its cross-talk with apoptosis was complex and deserved further investigation for better characterization.

In the study, we also successfully reduced PERK expression by intrathecal administration of the lentivirus vector in radiation myelitis banna pigs [10], [11] caused by ^125^I brachytherapy, the apoptosis rates were significantly higher than that in control group and deteriorated radiation myelitis of banna pigs, which was consistent with that in neural cells. In conclusion, autophagy caused by ^125^I radiation was mainly as an attempt of cell survival at an early stage, and it would be a self-destructive process and promoted the process of apoptosis and necrosis radiated by ^125^I for more than 72 hours. It would be useful and helpful to maximize efficiency of radiation therapy in clinical therapy.
